# Experimentally Induced Pulpal Lesion and Substance P Expression: Effect of Ketoprofen—A Preliminary Study

**DOI:** 10.1155/2016/6820781

**Published:** 2016-02-29

**Authors:** Gian Marco Abbate, Paola Sacerdote, Giada Amodeo, Alessandro Mangano, Luca Levrini

**Affiliations:** ^1^Faculty of Dentistry and Oral Hygiene, University of Insubria, Via Piatti 10, 21100 Varese, Italy; ^2^Dipartimento di Scienze Farmacologiche e Biomolecolari, University of Milan, 20129 Milano, Italy

## Abstract

*Objectives.* To evaluate substance P (SP) and the effect of ketoprofen administration, a nonsteroidal anti-inflammatory drug (NSAID), on SP in the pulp of upper third molars with experimentally induced pulpal lesion.* Materials and Methods.* A sample of 20 young systemically healthy adults of both sexes, nonsmokers, with a healthy upper third molar to extract for orthodontic purposes, was selected. Prior to the procedure, an inflammatory process was generated by mechanical exposure of the pulp. After 15 minutes, the pulp was collected using a sterile barbed broach. SP levels were determined by using a commercially available enzyme immunoassay (ELISA) kit. The subjects were randomly divided into two groups: group 1 received a dose of ketoprofen 30 minutes prior to the experimental procedure. The subjects of group 2 did not receive any kind of drug administration. The patients were asked to complete a diary on the postoperative pain.* Results.* No statistically significant difference could be detected in SP expression between the two groups. In group 1, pain manifestation was significantly delayed in comparison with group 2.* Conclusions.* Preventive administration of ketoprofen did not significantly affect the pulpal levels of SP but resulted in a significantly postponed manifestation of pain after extraction.

## 1. Introduction

Almost all pathological conditions that affect the oral tissues, as well as dental care or orthodontic procedures, increase the production and release of substance P (SP), a neuropeptide produced in a subset of capsaicin-sensitive sensory peripheral neuron cell bodies localized in the dorsal root and trigeminal ganglia [1]. This neuropeptide plays a pivotal role in the transmission of noxious stimuli in the spinal cord and the stimulation of capsaicin-sensitive sensory peripheral terminal of the neurons results in the peripheral release of several neuropeptides, including SP [2–4]. Evidence strongly supports the major role of SP in the development and maintenance of dental pain and inflammation [5]. Previous findings have demonstrated how SP represents a key neuropeptide in the generation of neurogenic inflammation, as in presence of caries [6], occlusal trauma [7], after cavity preparation [8], dentin-bonding agents application [9], after the application of tooth bleaching products [10], and in orthodontic movements [11, 12]. Several studies have shown that SP expression is significantly increased in the inflamed pulp, suggesting that SP plays a key role in the pulpal inflammatory process [13–15]. Moreover, Sattari and coworkers in 2010 [15] suggested that the presence of increased SP levels in the pulps of symptomatic teeth may open new directions for treating pulpal inflammation and pain. This study aimed to measure the SP expression and to evaluate the effect of ketoprofen administration, a nonsteroidal anti-inflammatory drug (NSAID), on the quantity of SP in the pulp of upper third molars with experimentally induced pulpal lesion.

## 2. Material and Methods

A sample of 20 young (19–30 years old) systemically healthy adults of both sexes, nonsmokers, was selected among the patients of the Department of Orthodontics of the University of Insubria, Varese, Italy. Inclusion criteria comprehended the presence of a healthy upper third molar programmed for extraction for orthodontic purposes. Written informed consent was obtained from each patient. The study was performed according to the recommendations regarding ethical issues in research with human tissues of the University of Insubria of Varese, Italy, and was conducted in accordance with the Declaration of Helsinki.

Prior to extraction, the teeth were anaesthetized (1.8 mL of 4% articaine with 1 : 100.000 epinephrine by infiltration injection) and isolated with a rubber dam. Shortly after a pulpal inflammatory process was generated by mechanical exposure of the pulp using a No. 1 round carbide bur mounted on a high-speed handpiece, the bur was used on the tooth without irrigation inducing also a thermal trauma in the underlying pulpal tissue. After a period of 15 minutes, the pulp was extracted using a sterile barbed broach. All pulp samples were placed into test tubes and kept frozen at −20°C until the SP expression determination.

The subjects were randomly divided into two groups: group 1 received a single dose of 80 mg ketoprofen lysine salt (the commercial name is “Oki” manufactured by Dompe CPA, Italy) in the form of sachet to dissolve in water, 30 minutes prior to the experimental procedure. The subjects of group 2 did not receive any kind of drug administration. After the extraction of the third molar, the wound was sutured and the patient was asked to complete a diary on the postoperative pain until the control appointment seven days after, in which time of appearance and recurrence of pain, necessity of use ketoprofen, and efficacy of the drug in controlling pain were analysed.

A verbal pain intensity scale was used, where pain intensity was indicated as follows: no pain, slight, moderate, intense, and severe [16]. Before the surgery, the patients were carefully instructed on the compilation of the diary and were further interviewed at the control appointment about the modalities of compilation in order to check the effectiveness of pain records.

For SP determination, pulp samples were weighed and 0.5 mL of acetic acid 0.5 N, containing a protease inhibitor cocktail (Roche, Milano, Italy), was added to all samples that were then boiled for 10 minutes. Samples were homogenized and centrifuged at 10000 ×g for 10 minutes at 4°C. Supernatants were collected, dried with a Speed Vacuum Concentrator, and resuspended in 0.3 mL of assay buffer. SP levels were determined by using a commercially available enzyme immunoassay (ELISA) kit according to the manufacturer's instructions (R&D system, Milano, Italy). Sensitivity of the kit was 15 pg/mL.

Nonparametric Mann-Whitney *U* test was applied to assess differences in the SP levels and to analyse differences in pain duration.

## 3. Results

The experimental procedure was completed with some complications, rubber dam resulted in being quite difficult to position in 6 patients (2 in group 1: patients 4 and 8; 4 in group 2: patients 3, 6, 7, and 9) and it was possible to fix it only with composite resin. Two pulp samples in group 1 (7 and 10) resulted in being difficult to analyse because of the exiguous quantity of pulp tissue collected. These two samples were not comprehended in the statistical analysis.

SP concentrations, expressed as pg/mg of pulp tissue, of each patient are reported in Tables 1 and 2. In the ketoprofen group, a higher variability was observed. As reported in Table 3, no statistically significant difference (Mann-Whitney *U* test) was present between the two groups. In Tables 1 and 2, also appearance of pain after extraction (expressed in hours), intensity of pain (classified by patients according to the following: no pain, slight, moderate, and intense pain), time of reduction of pain after ketoprofen intake (expressed in minutes) for the patients that felt necessary taking it, and the duration of pain relief (expressed in hours) are reported.

In group 1, pain appearance was significantly delayed (6.2 ± 0.13 hours) in comparison to patients who did not receive ketoprofen before the experimental procedure (3.95 ± 0.2 hours) (Table 3). This difference resulted in being statistically significant (*p* = 0.0002 Mann-Whitney *U* test). Generally, the patients experienced only slight to moderate pain; one patient described pain as intense. Only half of the patients in group 1 felt the necessity to take a second ketoprofen dose after tooth extraction, while in group 2 all the patients decided to take the drug at the appearance of pain after the surgical procedure.

## 4. Discussion

This study investigated the expression of substance P (SP) and the effect of ketoprofen administration, a commonly used nonsteroidal anti-inflammatory drug (NSAID), on the quantity of SP in the pulp of upper third molars with experimentally induced pulpal lesion. The approach of investigating SP levels in pulps with induced lesion could represent a possibility to standardize the experimental procedure in order to evaluate the effect of the NASID tested in presence of lesions with similar features and similar time outline. In the patients of group 1, a single dose of ketoprofen lysine salt (“Oki,” Dompe CPA, Italy) was administered 30 minutes prior to the experimentally induced pulpal lesion and the extraction of the third molar while in group 2 no drug was administered before the procedure. No statistically significant difference in SP expression could be detected between the two groups; only a higher variability was observed in the ketoprofen group.

Several studies have reported an increase in SP levels in inflamed pulps. Sattari and coworkers [15] reported no statistical differences in SP expression between pulp tissues with symptomatic and asymptomatic irreversible pulpitis confirming that there was a significant increase in neuropeptide expression in both inflammatory phenomena. Similarly, Caviedes-Bucheli et al. [17, 18] reported a significant SP increase in both inflamed pulps and irreversible pulpitis. In the present study, the SP levels in noninflamed pulp were not evaluated. However, the study of Caviedes-Bucheli et al. performed in 2006 [17] assessed the SP concentration with a comparable method with the one used in the present study and the SP expression in the healthy pulp resulted in 0.33 ± 0.2 pg/mg pulp. As expected, the values of SP here measured (87.33 ± 17 and 104 ± 10) are higher than those reported in the healthy pulp although they do not reach the levels that these authors measured in irreversible pulpitis (154 ± 52) [17]. These data confirm that an induced pulpal lesion seems to be enough to start inflammation in the pulp after 15 minutes, but probably this time is not sufficient for the inflammation process to fully take place and actually no data are present to evaluate exactly the time necessary for the completion of irreversible pulpitis. Moreover, local anaesthetics with vasoconstrictor (4% articaine with 1 : 100.000 epinephrine was used in the present study) seem to attenuate SP expression by the effect of alpha-adrenergic agonists as stated in different studies [19, 20]; actually Caviedes-Bucheli and coworkers [17, 20] used prilocaine without vasoconstrictor and that could also help to explain the difference reported with the data here presented.

The presence of increased SP levels may open new directions for treating pulpal inflammation and pain but no studies have yet been performed to evaluate the effect of common used NSAIDs on this parameter and, as far as we know, the present is the first study to address this aspect. Pain after extraction occurred significantly later in the group in which the NSAID was administered before the experimental procedure; the overall experience of pain in this group was described as better and only half of the patients decided to use painkillers after the extraction, while all the patients of the group with no drug administration prior to extraction reported intake 3–5 hours after the experimental procedure. Still, these data are not sufficient to configure a recommendation regarding the opportunity of ketoprofen administration before tooth extraction procedures.

In this preliminary study, considering the limited number of patients, ketoprofen does not seem to interfere significantly in the SP expression in the pulp with induced lesion. Considering the efficacy of this molecule in the modulation of pain and inflammation in third molar surgery previously reported [21], this result may appear rather disappointing. However, the short duration of the experimentally induced inflammation (15 minutes) may be responsible for the negative result and more studies with larger samples could be necessary to evaluate the effect of this molecule and other NSAIDs also in noninduced experimental pulpal lesions.

## 5. Conclusions

The study reported an increase in SP expression in the pulp of third molars with experimentally induced lesion, correlating with the data of previous studies. The presence of increased SP levels may represent a possibility for treating pulpal inflammation and pain. Pain after extraction occurred significantly later in the group in which ketoprofen was administered before the experimental procedure; still preventive administration of the drug did not significantly affect the pulpal levels of SP. More studies with other molecules and performed on larger samples are necessary to evaluate the effect of NSAIDs on SP expression.

## Figures and Tables

**Table 1:** Substance P and pain evaluation after tooth extraction of upper third molars with experimentally induced pulpal lesion, in patients with ketoprofen administration 30 minutes before the procedure.

Patients group 1	SP pg/mg pulp	Appearance of pain (hours after extraction)	Intensity of pain	Onset to pain relief (minutes after ketoprofen intake)	Duration of pain relief (hours)
1	158	7	Slight	No drug	
2	125.75	6	Moderate	30	>5
3	140.58	6	Moderate	30	>5
4	40.65	6	Slight	No drug	
5	88.9	7	Slight	30	>5
6	31.95	6	Slight	No drug	
7	8,23	6	Moderate	No drug	
8	72.53	6	Moderate	30	4-5
9	44.28	6	Slight	No drug	
10	9.33	6	Moderate	30	3-4

**Table 2:** Substance P and pain evaluation after tooth extraction of upper third molars with experimentally induced pulpal lesion, in patients with no drug administration before the procedure.

Patients group 2	SP pg/mg pulp	Appearance of pain (hours after extraction)	Intensity of pain	Onset to pain relief (minutes after ketoprofen intake)	Duration of pain relief (hours)
1	121.57	3	Moderate	30	>5
2	187.57	4,5	Slight	30	>5
3	83.43	4	Moderate	30	3-4
4	93.44	5	Moderate	30	4-5
5	73.25	5	Slight	30	>5
6	93.11	3	Slight	30	3-4
7	100.93	4	Moderate	30	>5
8	83.4	3,5	Slight	30	>5
9	88.44	3,5	Slight	30	3-4
10	122.71	4	Intense	30	4-5

**Table 3:** Pulp substance P levels and time to appearance of pain.

	Group 1 (ketoprofen)	Group 2 (no drug)	Mann-Whitney *U* test
SP pg/mg pulp (mean ± SEM)	87.33 ± 17 *n = 8*	104 ± 10.49 *n = 10*	*p = 0,45*

Appearance of pain (hours) (mean ± SEM)	6.2 ± 0.13 *n = 10*	3.95 ± 0.2 *n = 10*	*p = 0,0002*

## References

[1] Seybold V. S. (2009). The role of peptides in central sensitization. *Handbook of Experimental Pharmacology*.

[2] Maggi C. A. (1995). Tachykinins and calcitonin gene-related peptide (CGRP) as co-transmitters released from peripheral endings of sensory nerves. *Progress in Neurobiology*.

[3] White D. M. (1997). Release of substance P from peripheral sensory nerve terminals. *Journal of the Peripheral Nervous System*.

[4] Harrison S., Geppetti P. (2001). Substance P. *The International Journal of Biochemistry & Cell Biology*.

[5] Sacerdote P., Levrini L. (2012). Peripheral mechanisms of dental pain: the role of substance P. *Mediators of Inflammation*.

[6] Rodd H. D., Boissonade F. M. (2000). Substance P expression in human tooth pulp in relation to caries and pain experience. *European Journal of Oral Sciences*.

[7] Caviedes-Bucheli J., Azuero-Holguin M. M., Correa-Ortiz J. A. (2011). Effect of experimentally induced occlusal trauma on Substance P expression in human dental pulp and periodontal ligament. *Journal of Endodontics*.

[8] Caviedes-Bucheli J., Correa-Ortíz J. A., García L. V., López-Torres R., Lombana N., Muñoz H. R. (2005). The effect of cavity preparation on substance P expression in human dental pulp. *Journal of Endodontics*.

[9] Caviedes-Bucheli J., Correa-Ortiz J. A., Ballestero A. C. (2010). The effect of dentine-bonding agents on substance P release in human dental pulp. *International Endodontic Journal*.

[10] Caviedes-Bucheli J., Ariza-García G., Restrepo-Méndez S., Ríos-Osorio N., Lombana N., Muñoz H. R. (2008). The effect of tooth bleaching on substance P expression in human dental pulp. *Journal of Endodontics*.

[11] Chavarría-Bolaños D., Martinez-Zumaran A., Lombana N., Flores-Reyes H., Pozos-Guillen A. (2014). Expression of substance P, calcitonin gene-related peptide, *β*-endorphin and methionine-enkephalin in human dental pulp tissue after orthodontic intrusion: a pilot study. *The Angle Orthodontist*.

[12] Levrini L., Sacerdote P., Moretti S., Panzi S., Caprioglio A. (2013). Changes of substance P in the crevicular fluid in relation to orthodontic movement preliminary investigation. *The Scientific World Journal*.

[13] Bowles W. R., Withrow J. C., Lepinski A. M., Hargreaves K. M. (2003). Tissue levels of immunoreactive substance P are increased in patients with irreversible pulpitis. *Journal of Endodontics*.

[14] Caviedes-Bucheli J., Azuero-Holguin M. M., Munoz H. R. (2005). The effect of capsaicin on substance P expression in pulp tissue inflammation. *International Endodontic Journal*.

[15] Sattari M., Mozayeni M. A., Matloob A., Mozayeni M., Javaheri H. H. (2010). Substance P and CGRP expression in dental pulps with irreversible pulpitis. *Australian Endodontic Journal*.

[16] Williamson A., Hoggart B. (2005). Pain: a review of three commonly used pain rating scales. *Journal of Clinical Nursing*.

[17] Caviedes-Bucheli J., Lombana N., Azuero-Holguín M. M., Munoz H. R. (2006). Quantification of neuropeptides (calcitonin gene-related peptide, substance P, neurokinin A, neuropeptide Y and vasoactive intestinal polypeptide) expressed in healthy and inflamed human dental pulp. *International Endodontic Journal*.

[18] Caviedes-Bucheli J., Gutierrez-Guerra J. E., Salazar F., Pichardo D., Moreno G. C., Munoz H. R. (2007). Substance P receptor expression in healthy and inflamed human pulp tissue. *International Endodontic Journal*.

[19] Pertl C., Amann R., Odell E., Robinson P. D., Kim S. (1997). Effects of local anesthesia on substance P and CGRP content of the human dental pulp. *Journal of Endodontics*.

[20] Caviedes-Bucheli J., Rojas P., Escalona M. (2009). The effect of different vasoconstrictors and local anesthetic solutions on substance P expression in human dental pulp. *Journal of Endodontics*.

[21] Pouchain E. C., Costa F. W., Bezerra T. P., Soares E. C. (2015). Comparative efficacy of nimesulide and ketoprofen on inflammatory events in third molar surgery: a split-mouth, prospective, randomized, double-blind study. *International Journal of Oral and Maxillofacial Surgery*.

